# The evolution of the axial skeleton intercentrum system in snakes revealed by new data from the Cretaceous snakes *Dinilysia* and *Najash*

**DOI:** 10.1038/s41598-018-36979-9

**Published:** 2019-02-04

**Authors:** Fernando F. Garberoglio, Raúl O. Gómez, Tiago R. Simões, Michael W. Caldwell, Sebastián Apesteguía

**Affiliations:** 1 CONICET, Buenos Aires, Argentina; 2 Área de Paleontología, Fundación de Historia Natural Félix de Azara. CEBBAD, Universidad Maimónides. Hidalgo 775 (1405), Buenos Aires, Argentina; 3 IGEBA-Departamento de Ciencias Geológicas, Facultad de Ciencias Exactas y Naturales, Universidad de Buenos Aires, Ciudad Universitaria (1428), Buenos Aires, Argentina; 4 Departamento de Biodiversidad y Biología Experimental, Facultad de Ciencias Exactas y Naturales, Universidad de Buenos Aires, Ciudad Universitaria (1428), Buenos Aires, Argentina; 5 Department of Biological Sciences, University of Alberta, Edmonton, AB T6G 2E9 Canada; 6 Department of Earth and Atmospheric Sciences, University of Alberta, Edmonton, AB T6G 2E9 Canada

## Abstract

Snakes are an extremely modified and long-lived clade of lizards that have either lost or highly altered many of the synapomorphies that would clearly link them to their closest sister-group among squamates. We focus here on one postcranial morphological complex, the intercentrum system which in most non-ophidian squamates is limited to the cervical and caudal regions. The Cervical Intercentrum System (CeIS) is composed of a single intercentral element that sometimes articulates with a ventral projection (hypapophyses) of the centrum; the Caudal Intercentrum System (CaIS) is formed by an intercentral element, the haemal arch/chevron bone, and paired ventral projections of the centrum, the haemapophyses. In modern snakes, the intercentrum element of the CeIS is considered lost or fused to the hypaphophysis, and the chevron bone in CaIS is considered lost. Here, we describe new specimens of the early snake *Dinilysia patagonica*, and reinterpret previously known specimens of *Dinilysia* and *Najash rionegrina*, that do not show the expected snake morphology. The anatomy of these fossil taxa unambiguously shows that free cervical and caudal intercentra attached to distinct downgrowths (hypapophyses and haemapophyses) of the centra, are present in basal fossil snakes, and agrees with the proposed loss of post atlas-axis intercentra in later evolving snakes.

## Introduction

Modern snakes are an extremely diverse clade of lizards with an improving fossil record that is revealing unexpected data on a large number of problematic snake anatomical features, *i*.*e*., the presence of small to large rear limbs^1,2^, the otic region and organization of the crista circumfenestralis^3^, or the presence of a jugal, and the identity of the circumorbital bones^4^. Though the skull of snakes has been implicated as the major driver of snake evolution^5^ (against recent claims to the contrary^6^), the postcranial skeleton of these animals also displays problematic anatomies by comparison to that of other lizards, limbed or limbless, short bodied, or elongate.

**Figure 1:**
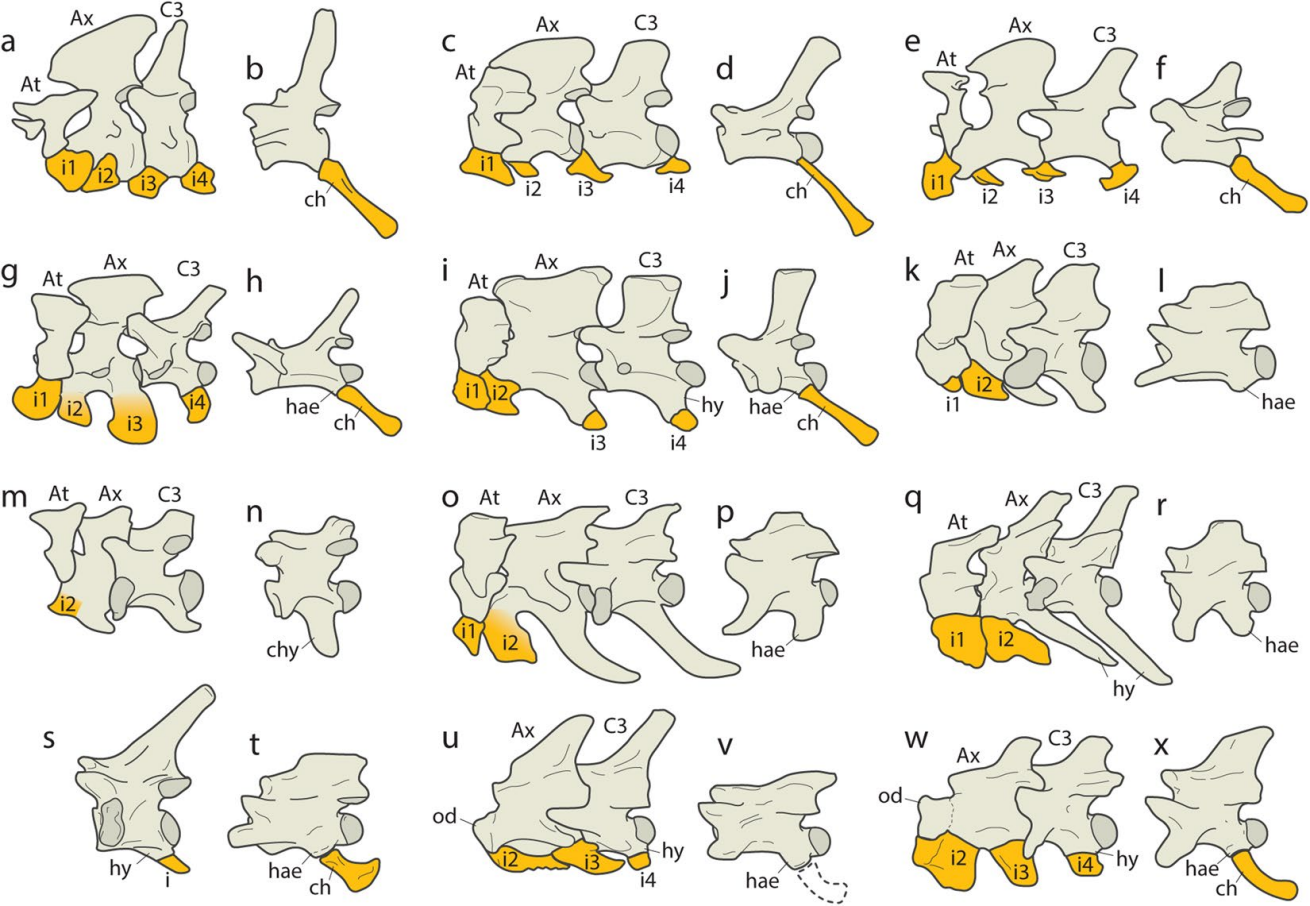
Survey of intercentrum system in selected lepidosaurs. (**a**) cervical vertebrae of *Sphenodon*; (**b**) caudal vertebra of *Sphenodon* UAMZ 405; (**c**) cervical vertebrae of *Iguana*; (**d**) caudal vertebra of *Iguana* MCZ 10975; (**e**) cervical vertebrae of *Rachodactylus*; (**f**) caudal vertebra of *Gekko* MCZ 173377; (**g**) cervical vertebrae of *Shinisaurus*; (**h**) caudal vertebra of *Shinisaurus*; (**i**) cervical vertebrae of *Varanus*; (**j**) caudal vertebra of *Varanus* USNM 287277; (**k**) cervical vertebrae of *Anilius* MCZ 19537; (**l**) caudal vertebrae of *Anilius* MCZ 19537; (**m**) cervical vertebrae of *Uropeltis* MCZ 3873; (**n**) caudal vertebra of *Rhinophis*; (**o**) cervical vertebrae of *Xenopeltis* USNM 287277; (**p**) caudal vertebra of *Xenopeltis* USNM 287277; (**q**) cervical vertebrae of *Python* FMNH 51631; (**r**) caudal vertebra of *Python* ZFMK 83431; (**s**) cervical vertebra of *Haasiophis* (based on HUJ-Pal EJ 695); (**t**) caudal vertebra of *Haasiophis* (based on HUJ-Pal EJ 695); (**u**) cervical vertebrae of *Najash* (based on MPCA-PV 391); (**v**) caudal vertebra of *Najash* (based on MPCA-PV 396); (**w**) cervical vertebrae of *Dinilysia* (based on MPCA-PV 527 and MACN-PV-N 116) and (**x**) caudal vertebrae of *Dinilysia* (based on MACN-RN-1016). Abbreviators: At, atlas; Ax, axis; C, cervical; ch, chevron bone; chy, caudal hypapophysis; hae, haemapophysis; hy: hypapophysis; i, intercentrum. For taxa without specimen number, source of data is from available literature: a, c, e, i, and n;^7^ g and h^49^).

One of these problematic postcranial features concerns the morphology of the intercentrum system of lizards, and how that system of extra-central vertebral bodies has been modified through not just lizard evolution, but more particularly, through snake evolution (Fig. 1). The classic review of the squamate intercentrum system^7^, while thorough and current for the time, only reviewed the osteology of extant squamates and thus characterized the higher level groups around only the anatomy of the modern forms. With the recent discovery of new specimens of previously known fossil snakes, *i*.*e*., *Dinilyisa patagonica*, and new taxa of fossil snakes, *i*.*e*., *Najash rionegrina*, there have been a number of recent studies explicating new data and interpretations of the snake intercentrum system, and more specifically the cervical and caudal intercentrum systems^8–11^.

In general terms^7^, in all squamates with the exception of gekkotans and xantusiids, the intercentrum system is limited to the cervical and caudal regions (Fig. 1). The cervical intercentrum system (CeIS) is composed of a single intercentrum element that in most squamates articulates with a variably positioned pedicle or peduncle of bone arising from the centrum surface that is referred to as the hypapophysis; positioning of the hypapophysis is either anterior on the centrum, posterior on the centrum, or at the inter-centrum contact so that the intercentrum body, is indeed ‘intercentral’. When the intercentrum contacts both the anterior and posterior centra at their articulation, it never fuses to either centra, though it may fuse when articulating with a rostral or caudal hypapophysis.

The caudal intercentrum system (CaIS) is somewhat different by comparison to the CeIS. The intercentrum proper forms a protective arch of bone, *i*.*e*., the haemal arch, around the deep arterial system of the caudal artery and associated vasculature and nerves. The haemal arch generally forms a Y-shaped bone, or chevron, the distal extent of which serves as a muscle and ligament attachment zone for the hypaxial musculature. The right and left centrum contact points for the forks of the chevron bone/haemal arch, are a pair of pedicles or haemapophyses. The haemapophyses are generally placed posteriorly on the caudal centrum, but are, in some cases, at the inter-central articulation, and in some squamates the chevron forks fuse to the haemapophyses. In squamates with specialized adaptations such as aquatic locomotion, the haemal arch spines can be distally elongated, in many cases, longer than the neural spines, in order to increase the lateral aspect of the tail for caudal propulsion, and increase the muscle mass used to generate force.

As noted, when first^7^ characterized, the snake CaIS and CeIS were described based on observations of modern snakes. The ‘cervical’ region of snakes has long been considered ‘lost’ and unrecognizable anatomically, because of the absence of the pectoral girdle as a key landmark^7^. Still, those authors did report on the presence, often in extremely large numbers and extending some distance posteriorly, of hypapophyses on the anterior precloacals of modern snakes; these ‘hypapophyseal processes’ begin on the axis centrum (just posterior to the one certain intercentrum body present in a modern snake, the atlas intercentrum).

Recent work, looking at the anatomies of both fossil and extant snakes, has made it clear that the neck of snakes can be demarcated based on both osteological and myological anatomies, such that snakes really do possess a neck and thus, diagnosable cervical vertebrae^9,11–13^. Such a distinction is not trivial as the neck/cervical region of snakes would be predicted, based on the fact that snakes are lizards, to display features of a CeIS and thus possess hypapophyses and intercentra. A HOX-gene expression study^14^ examining *Python*, concluded that thoracic identity had been overprinted onto the ‘neck’ region in snakes based on the presence of anterior ribs. A responding study^15^ noted that cervical ribs had not been reacquired in snakes (the morphological feature used^14^ to qualify thoracic identity), but rather are a primitive feature for all vertebrates including any clade that might be considered the sister-group of snakes. The presence of hypapophyses throughout much of the column of many moderns groups of snakes was cited as indicating that cervical identity, not thoracic identity, had dominated the development and evolution of the precloacal vertebrae in snakes^15^.

As a result, if “cervicalization” so to speak, not “thoracalization”, has dominated snake neck and thorax evolution (the precloacal vertebral column), it should come as no surprise that the cervical region of fossil snakes is easily demarcated, and that the cervical vertebrae of fossil snakes would reveal the presence of unfused intercentra articulating with hypapophyses in the anteriormost cervicals, but still posterior to the atlas-axis intercentra^9,11,12^. These fossil anatomies provide important information on the presence of intercentra in the anterior part of the column, the CeIS, at an early stage in snake evolution.

With respect to the CaIS, modern snakes do not possess a Y–shaped, or chevron–shaped haemal arch, but instead generally possess paired, ventrally directed haemapophyseal processes of the centrum^7,10^; these paired processes in snakes have been long interpreted to represent the intercentrum in some form or another even though they are classically referred to as ‘haemapophyses’. Clarity on this problem is revealed only by reference to the anatomies preserved in fossil snakes, where new specimens have been found with well-articulated caudal elements that include distinct haemapophyses and chevrons/intercentra: *Eupodophis descouensi*, *Haasiophis terrasanctus*, and *Wonambi naracoortensis*^11,16,17^. Despite the observed anatomies, the homology of the CaIS in these fossil taxa to the CaIS of other squamates has been challenged by some authors^10,18^ prior to the study of Palci *et al.*^11^.

The intent of this study is to report data on the CaIS and CeIS from new specimens of the fossil snake *Dinilysia patagonica* Woodward, 1901. Here, we will refer to the downgrowths of the centra that act as contact points for the cervical and caudal intercentral elements as hypapophyses and haemapophyses respectively, instead of using others terms that have been used in the literature to refer to these structures (e.g., pedicles, pedicels, peduncles, hypapophyses s.s.). A secondary goal is to synthesize these new data with existing data in fossil and modern snakes. We therefore re-characterize the previously described cervical series of *D*. *patagonica*^9^ and compare it to known specimens of *Najash rionegrina* and build a set of hypothesis regarding the evolution of the CaIS and CeIS in snakes against current phylogenies. We also describe the first known caudal series of the early snake *D*. *patagonica*, based on articulated remains from the Bajo de la Carpa Formation of the Neuquén Group (Santonian, Upper Cretaceous), Río Negro Province, Argentina. The specimen described herein comprises a string of 13 vertebrae, from posterior-most precloacals to the first caudals, displaying haemapophyses and articulated chevron bones. This presents unequivocal evidence for the presence of a CaIS in *Dinilysia patagonica*, and additional evidence of a CaIS in snakes that is homologous to that of other squamates. Comparisons between the CaIS in *Dinilysia* with other fossil snakes provides important insights on the presence of a CaIS in the early evolution of snakes and it leads to an important interpretation regarding the homology of the ventral projections of the caudal vertebrae among extant snakes (see also^11^).

## Results

### Cervical Intercentrum System (CeIS)

#### Re-characterization and new material of *Dinilysia patagonica*

**Figure 2:**
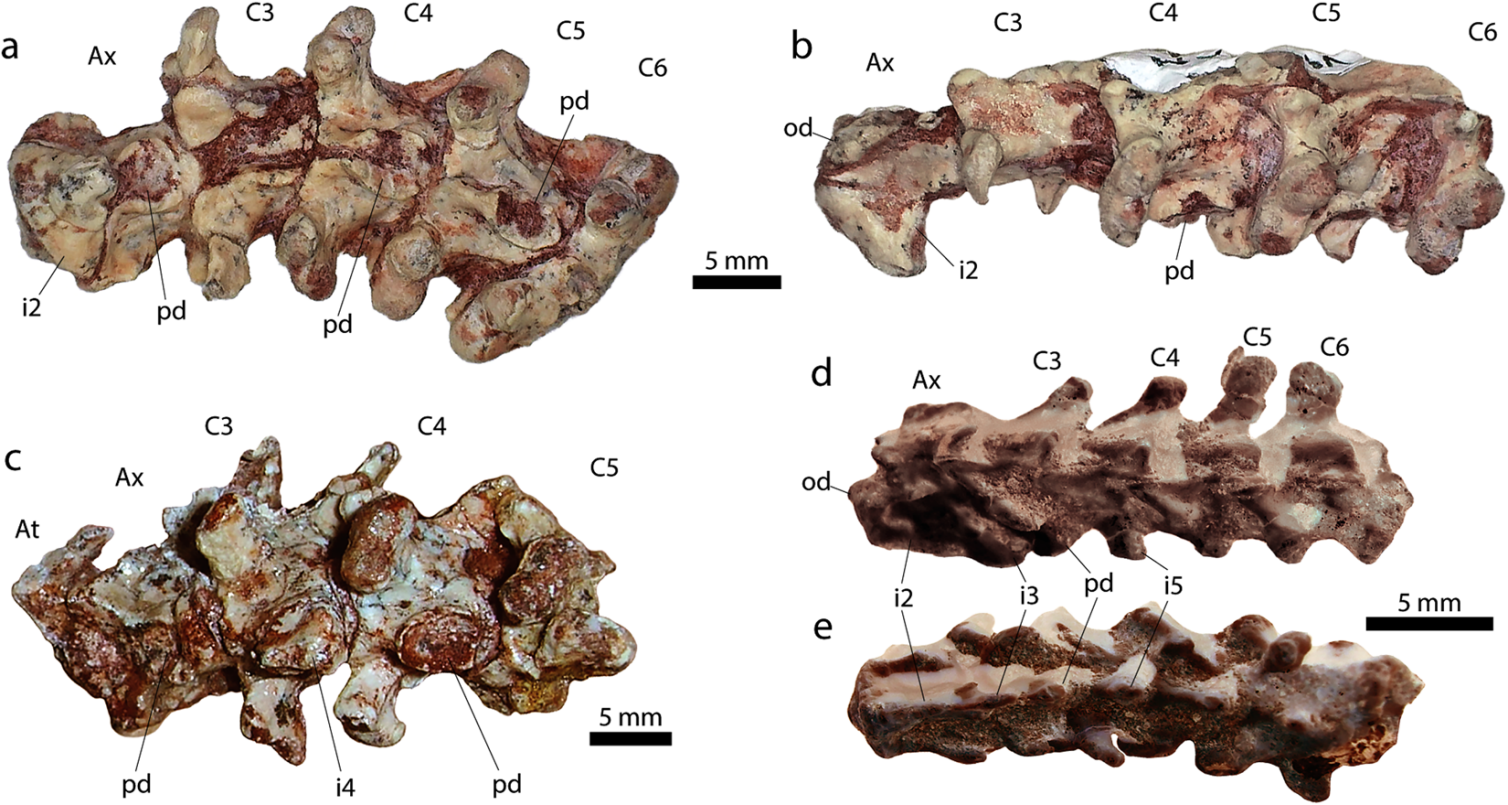
Cervical vertebrae of *Dinilysia patagonica* (**a–c**) and *Najash rionegrina* (**d**,**e**). MPCA-PV-N 116, ventral view (**a**) and lateral view (**b**). MPCA-PV 527, ventral view (**c**). MPCA-PV 391, lateral view (**d**) and ventral view (**e**). Abbreviators: At, atlas; Ax, axis; C, cervical; i, intercentrum; hy, hypapophysis; o, odontoid process.

Unsuspected vertebral features for the cervicals of the fossil snake *Dinilysia patagonica* were reported^8,9^ from a specimen of *Dinilysia* (MPCA-PV 527) collected at Paso Córdova in 2001 (*i*.*e*., a partial skull articulated to a series of five vertebrae [Fig. 2]). These authors purposefully referred to these five vertebrae as cervicals in full recognition of previous work on the subject (e.g.^1,7,15,19–21^). The reasoning used by the authors^8,9^ for choosing ‘cervical’ over ‘precloacal’ was straightforward: problems delimiting a ‘neck’ as a region along the body axis are distinct from the question of whether or not an anterior vertebra possesses osteological characters that identify it as a cervical vertebra. They argued that the classic criterion^7^ *i*.*e*., presence and position of the first dorsal vertebra with a rib articulating with the sternum, was a delimiter of ‘neck’, and we continue that logic here with the argument that ‘neck’ is not a synonym of ‘cervical’ and that there are osteologies diagnostic of cervical vertebrae in the absence of a ‘neck’. Previous authors^8,9^ rejected the term ‘precloacals’ in favour of the term ‘cervicals’, even though ‘cervicals’ are a subset of the ‘precloacal’ region in the absence of a defined ‘neck’.

In MPCA-PV 527, the atlas neural arches are small, though mostly still in articulation with the intercentrum; the neural spine is narrow and quite short, projecting slightly posteriorly. The atlas intercentrum is not well preserved and its articulation with the axis vertebra is difficult to clearly define. We agree^9^ that it is not clear if there is an articulating intercentrum and an axis hypapophysis preserved in this specimen. The third and fourth cervicals have large, posteriorly positioned hypapophyses with large, unfused intercentra (Fig. 2). The intercentrum of the fourth precloacal is not preserved, permitting observation of a deeply concave articulation surface of the hypapophysis. In MPCA-PV 527, in contrast to the more anterior precloacals, the fifth cervical bears a large hypapophysis, with a possible fused intercentrum, and may represent the fate of the intercentrum in all modern snakes, consistent with previous conclusions^7^ (but see below).

Another set of articulated vertebrae from *Dinilysia* (MACN-PV-N-116) is described here and are also referred to as cervicals and they preserve some aspects not visible in MPCA-PV 527 (Fig. 2). The intercentrum and neural arches of the atlas are not present in the five vertebrae preserved in MACN-PV-N-116. The axis bears a developed odontoid process and preserves two ventral projections; the first ventral projection, which is large and located beneath the suture of the odontoid process to the axis centrum, represents the second intercentrum. It can be seen that it is sutured to the axis and articulating directly to the centrum, but not by a distinct, raised, hypapophysis. The second ventral projection on the axis is located posteriorly, and forms the first hypapophysis, low and rounded; the corresponding free intercentrum (the third) is lost, which is inferred by the deeply concave surface on the hypapophysis. Although no intercentra are preserved in this specimen, other than the one on the axis, the concave surface of the hypapophysis suggests the articulation of a free intercentrum as seen in the known cervicals of *Dinilysia*. The fourth vertebra in MACN-PV-N-116 shows quite clearly the same condition that is present in MPCA-PV 527 (Fig. 2). The fifth vertebra in MACN-PV-N-116 bears a narrower and less-rounded hypapophysis that seems to have a similar concave surface to the preceding ones. There are no obvious signs of fusion of the intercentra to the hypapophyses as was proposed for MPCA-PV 527^9^. The sixth vertebra is broken and its condition cannot be ascertained.

#### Re-characterization of *Najash rionegrina*

The holotype of *Najash rionegrina* preserves parts of the cervical series (MPCA-PV 391, see^22^). The atlas neural arches are not preserved. As described^22^ the axis is elongated and bears an odontoid process and well-developed pleurapophyses. Both of the axis ventral projections are posteriorly oriented, and the second one bears on each side a characteristic hook-like lateral projection. As the condition of the axis is not well preserved in the holotype, *Najash* was inferred^22^ to show the usual condition in snakes (with the second intercentrum sutured and the third one fused to the axis) based on what is seen on another specimen from the same locality, MPCA-PV 383 (see^22^). Regardless of the condition of MPCA-PV 383 (which seem to show both intercentra sutured, or only partially fused), assignment to *Najash rionegrina* has been questioned (see^23^), and the holotype axis intercentra are most likely sutured to the axis. More important, subsequent description^22^ characterized the third and fourth vertebrae of *Najash* as possessing “rod-like” hypapophyses. In contrast, we interpret that the cervicals of *Najash rionegrina* possess free intercentra. The third vertebra is the one that shows this condition more clearly, where the intercentrum is preserved in place, articulated to the posteriorly positioned hypapophyses (Fig. 2), evidenced by the suture between the two elements. On the fourth vertebra the intercentrum is lost and the rounded hypapophysis shows a deep concave terminal surface as is seen in the cervicals of *Dinilysia*.

### Caudal Intercentrum System (CaIS)

#### New material of *Dinilysia patagonica*

**Figure 3:**
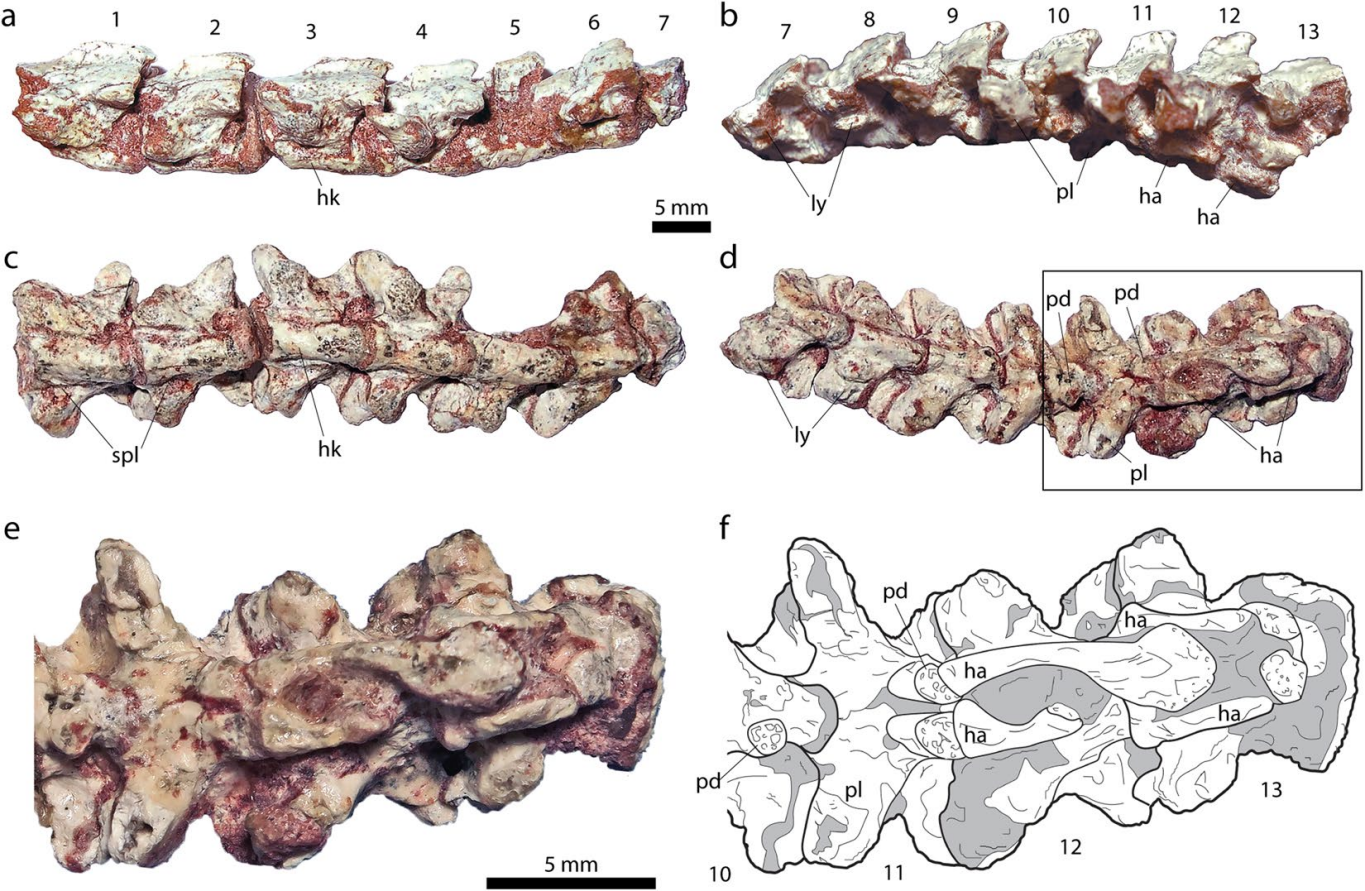
Caudal vertebrae of *Dinilysia patagonica*. MACN-RN-1016, lateral view (**a**,**b**), ventral view (**c**,**d**), detail of posterior ventral view (**e**), and interpretative drawing (**f**). Abbreviators: ha, haemal arch; hae, haemapophysis; hk, haemal keel; ly; lymphapophysis; pl, pleurapophysis; spl, subcentral paralymphatic fossae.

MACN-RN-1016 consists of a string of thirteen articulated vertebrae (Fig. 3). The anterior-most five vertebrae of the series show the typical morphology of posterior precloacals, *i*.*e*., a strong interzygapophyseal constriction that is more pronounced than in the middle trunk region^24^. They have well-developed parapophyses with a wide and low haemal keel that is anteriorly separated from the parapophyses by moderately deep depressions, and are pierced by subcentral paralymphatic foramina. None of these vertebrae show evidence of fused ribs as occurs in *Najash* and many non-ophidian squamates^7,23^. The three subsequent vertebrae (6^th^ to 8^th^) are somewhat different from the posterior most precloacals in having a narrower haemal keel and lacking the smaller and more ventrally oriented parapophyses. Additionally, the 7^th^ and 8^th^ vertebrae preserve lymphapophyses (although only their bases are preserved) that have a ventrally directed branch and a dorsal branch, directed laterally, marking the cloacal region of the series. The 6^th^ vertebra of the series is too badly preserved to be properly identified and it could represent either a cloacal or possibly a sacral. The configuration of the lymphapophyses resembles that of madstoiids from Patagonia^25^ (ROG per. obs.) and contrasts with that of scolecophidians, which have the ventral branch of the lymphapophyses more laterally oriented^26^. Additionally, alethinophidians typically have three to five vertebrae with lymphapophyses, whereas scolecophidians range from two to six^11,26^. The type specimen of *Najash* has been described^22^ as possessing three caudals bearing lymphapophyses, but that description has been contested^23^ with the argument that the first of these vertebrae is better interpreted as a sacral.

The succeeding vertebrae (9^th^ to 13^th^) are regarded as postcloacals based on the presence of pleurapophyses along with a poorly defined haemal keel or lack of it, and, in all except the first postcloacal, haemapophyses (Fig. 3e,f). The anterior postcloacals display distinct posteroventral haemapophyses with a distally articulated chevron. The 10^th^ vertebra preserves only one haemapophysis close to the condylar constriction and lacks an articulated chevron. The following caudals (11^th^ and 12^th^) have haemapophyses articulating with chevrons preserved in place that consist of paired and elongate arches (Fig. 3e,f).

The paired haemal arches are slightly curved inward and are sutured to a blunt and rounded haemal spine. The distal porous surface of the haemal spine suggests that it was capped by cartilage as in anguimorphan lizards (e.g. *Heloderma*, FFG pers. obs.). The relatively short chevrons of *Dinilysia* do not show a long and slender haemal spine, such as the Y-shaped chevrons of aquatic *Eupodophis*^11^, which is expected in a terrestrial form^27^, but are longer than the remarkably short chevrons of the marine snake *Haasiophis*^11^. The pleurapophyses are badly damaged in most postcloacals of MACN-RN-1016, but in a few vertebrae they are preserved as blade-like slightly anteriorly oriented processes, partly resembling the pleurapophyses of *Najash*^22,23^. An isolated postcloacal vertebra referred to *Diablophis gilmorei* also shows a pleurapophysis similar in size to MACN-RN-1016, although more posteriorly oriented^5^. The neural spines of the caudals are higher than those of posterior precloacals as in *Najash*^22,23^. As mentioned above, the first postcloacal (9^th^ vertebra) lacks haemapophyses and thus can be regarded as a pygal along with the cloacals (caudals lacking haemapophyses). The presence of at least one pygal is well documented in squamates^7^, including some fossil snakes, such as *Wonambi barriei*^17,28^. In *Najash*, the first caudals that bear pleurapophyses are not visible on the ventral side^22,23^.

#### Re-characterization of *Najash rionegrina*

The cloacal region of *Najash rionegrina* was recently re-described^23^. We agree with the new description (see^23^) which states that the vertebra that was originally identified^2,22^ as the first caudal with lymphapophyses is actually the only sacral vertebra, and that the two sacrals originally identified^2,22^ are better interpreted as presacral vertebrae bearing fused ribs. A more conservative approach^23^ recognized two or more vertebrae bearing lymphapophyses following the sacral, as the ventral side is not visible in that region. But, from the third caudal, the vertebrae show processes that are laterally and anteroventrally directed and are better interpreted as pleurapophyses^22^. *Najash* has also been described as having non-articulating blunt ‘haemapophyses’ like those of *Anilius*^2,22^. However, the short blunt ventral processes observed in the caudals of *Najash*, bearing semi-porous bone on the flat distal surface, are very similar to the condition reported herein for *Dinilysia*. Therefore, these processes in *Najash rionegrina* are re-interpreted as haemapophyses, and it is quite likely that the blunt flat distal ends of those processes would have articulated with chevrons in life (not preserved in the available specimens). The first two caudals, bearing lymphapophyses, lack any trace of haemapophyses. The third and fourth are not visible on the ventral side, and the fifth caudal already shows haemapophyses, so it is not possible to ascertain how many pygals were present.

## Discussion

In the CeIS, free cervical intercentra are not known in living snakes, apart from those present in the atlas-axis complex. The only vertebra that retains a certain free intercentrum in modern snakes is the atlas. The first vertebra, except in the Uropeltidae, is formed by the neural arches and an intercentrum, as the atlas centrum is incorporated into the axis forming the odontoid process^7^. The axis of modern snakes presents two ventral projections and the common condition is that the anterior of these two is sutured to the axis, although it can be fused (e.g., in most uropeltids; *Xenopeltis*), while the posterior ventral process is always fused^19,22^. According to Hoffstetter and Gasc^7^, these ventral projections represent the second and third intercentrum, and the remaining post-axial ‘hypapophyses’ are composed of fused intercentral elements. Other authors, based on embryological evidence^29^, consider the intercentra to be lost beyond the atlas-axis complex in modern snakes. The development of trunk vertebrae in macrostomatan snake embryos shows that the post-axis ventral projections are the result of a distal spreading-ossification of the pleurocentra^29^.

At the time of description of MPCA–PV 527^9^, no other fossil or extant snake was known to possess hypapophyses with unfused intercentra and a concave articulation surface on the hypapophysis (see^11^ for a list of new fossils snakes with hypapophyses and intercentra). It was noted^9^ that on the axis of *Yurlungurr camfieldensis* (see^19^) there is a large elliptical concavity on the distal tip of the hypapophysis. Also, the first axis intercentrum in *Yurlunggur* is likely sutured, or only partially fused to the axis centrum^19^. In the axis of *Dinilysia*, both of the intercentra are sutured, the second directly to the centrum and the third to the first hypapophysis. The posterior position of the hypapophyses in *Dinilysia*, *Najash*, potentially *Yurlungurr*, and other madtsoiids^25^, coupled with the presence of unfused intercentra are otherwise only observed in mosasaurs^30^, dolichosaurs^31^, pontosaurs^32^, and adriosaurs^21^. Varanoid lizards have often been compared to mosasaurs, but we have presented data here that indicates that the condition in *Najash* and *Dinilysia* is more similar to mosasaurs while varanids are more similar to modern snakes. In *Varanus*, the hypapophysis is not a rounded to ovate pedestal raised slightly above the body of the centrum and bearing an ovate depression at the tip. Rather, it is a long, thin, stalk-like structure, to which a single, or sometimes paired, “supposed”^7^ intercentrum element bearing extra epiphyseal chondrifications is attached at its distal tip.

The condition of the cervical vertebrae of *Dinilysia* and *Najash* indicates the presence in basal snakes of free unfused cervical intercentra articulating with a ventral posterior hypapophyses posterior to the axis. This implies, as the developmental evidence shows, that the intercentrum is lost beyond the atlas-axis complex in macrostomatans^29^, and that the ‘hypapophyses’ of macrostomatans snakes can be considered homologous to the proper hypapophyses present in fossil snakes and other non-ophidian squamates. However, developmental evidence of the condition in modern non-macrostomatan snakes is lacking, and the cervical series of *Dinilysia* shows a possible fusion of the intercentra to the posterior cervical hypapophyses^9^. While ‘apparent’ complete loss of the intercentra in later evolving snakes could have been accomplished by fusion of the intercentra to the hypapophyses, there is no evidence to support such a fusion, and thus simple loss (*i*.*e*., fate of the intercentra is not early stage fusion, but rather loss of the structure altogether) is supported by the evidence currently available. Moreover, because the first ventral element on the axis of *Dinilysia*, *Najash*, and possibly *Yurlunggur*^19^, is sutured to the centrum of the axis, without development of a hypapophyses, this first ventral element on the axis of modern snakes can be regarded as only the second intercentrum. This indicates that the first axis intercentrum becomes directly fused to the centrum in some extant taxa (although kept sutured in most extant taxa) (Fig. 1),while the remaining intercentra are likely lost and only the hypapophyses remain.

In the CaIS of most extant snakes, the postcloacal vertebrae present ventral projections of the caudal centra that are usually bifid distally and almost never contact each other. Some exceptions can be found, however, among males of some extant colubrine snakes (in which the distal tips of these processes may be more elongate and bent inwards contacting at the midline^33^) or in some sea-snakes (hydrophiines), where the haemapophyses can be very elongated and sometimes fused distally forming a complete arch^34^. On the other hand, there are instances in which only a single process is observed in the postcloacal vertebrae (e.g.: uropeltids^7^; most hydrophiines^34^). These structures have been generally termed ‘haemapophyses’, and they have been considered to represent the fusion of the caudal intercentra to the vertebral pleurocentra in snakes^7,10^. However, others consider that extant snakes have lost the intercentra throughout the vertebral column (with the exception of the atlas-axis complex) and the haemapophyses are actually downgrowths of the vertebral centrum and therefore homologous to the haemapophyses of lizards (e.g.^11,29^). The presence of unfused postcloacal intercentra (true chevrons) articulating with ventral projections of the centrum body (haemapophyses) in *Dinilysia*, suggests that the ‘haemapophyses’ of extant snakes are actually homologous to those of other squamates, with chevron elements being entirely lost. This is also supported by the typically paired nature of those processes in most extant snakes, since haemapophyses are also paired in all squamates (when present), whereas chevron bones usually form a single fused element.

Paired posteroventral processes similar to the haemapophyses of *Dinilysia* have been previously reported in other fossil snakes, namely *Najash* and madtsoiids, but these were interpreted in different ways. These were either described as typical derived ‘haemapophyses’ like those of extant snakes (considered as intercentra fused to caudal centra^2,22,35–38^), or as true haemapophyses, such as those of many non-ophidian squamates^17,25,39–41^. The new specimen reported here confirms the observations of the latter set of authors.

To date, indications of a full CaIS in terrestrial snakes were restricted to a single isolated vertebra bearing a chevron bone referred to *Wonambi*^17^, and in the marine simoliophiids *Eupodophis* and *Haasiophis*^11,16^. However, the condition in *Wonambi* was questioned by Rieppel *et al*.^38^, who interpreted the posteroventral elements on the cloacal vertebra of madtsoiids as ‘haemapophyses’ not-articulating with chevrons. Similarly, *Najash* has also been described as having non-articulating blunt ‘haemapophyses’^2,22^. However, as re-described here, the ventral processes observed in *Najash* present distal articulatory surfaces, likely for chevrons that are not preserved. This is supported by the presence of chevrons in the haemapophyses of *Dinilysia* that share the same morphological features with the type of haemapophyses found in *Najash* and madstoiids.

The CaIS of the marine simoliophiids *Eupodophis* and *Haasiophis*^11,16^ are very different from each other and also contrast with that of *Dinilysia*. The short, spatula-shaped chevrons of *Haasiophis* also articulated with posteroventral haemapophyses, but they are very short and posteriorly directed, whereas those of *Eupodophis* are seemingly attached directly to the posteroventral edge of the centrum^11^, and are much more elongate than the chevrons in *Haasiophis* and *Dinilysia*. It is noteworthy that the homologies of the caudal structures of these marine snakes, hindered in part by the peculiar morphology and preservation issues of the fossils, have also been hotly debated, being interpreted as true chevrons (e.g.^11,16^) or as neomorphic structures with no homologies to the CaIS (e.g.^10,42^).

**Figure 4:**
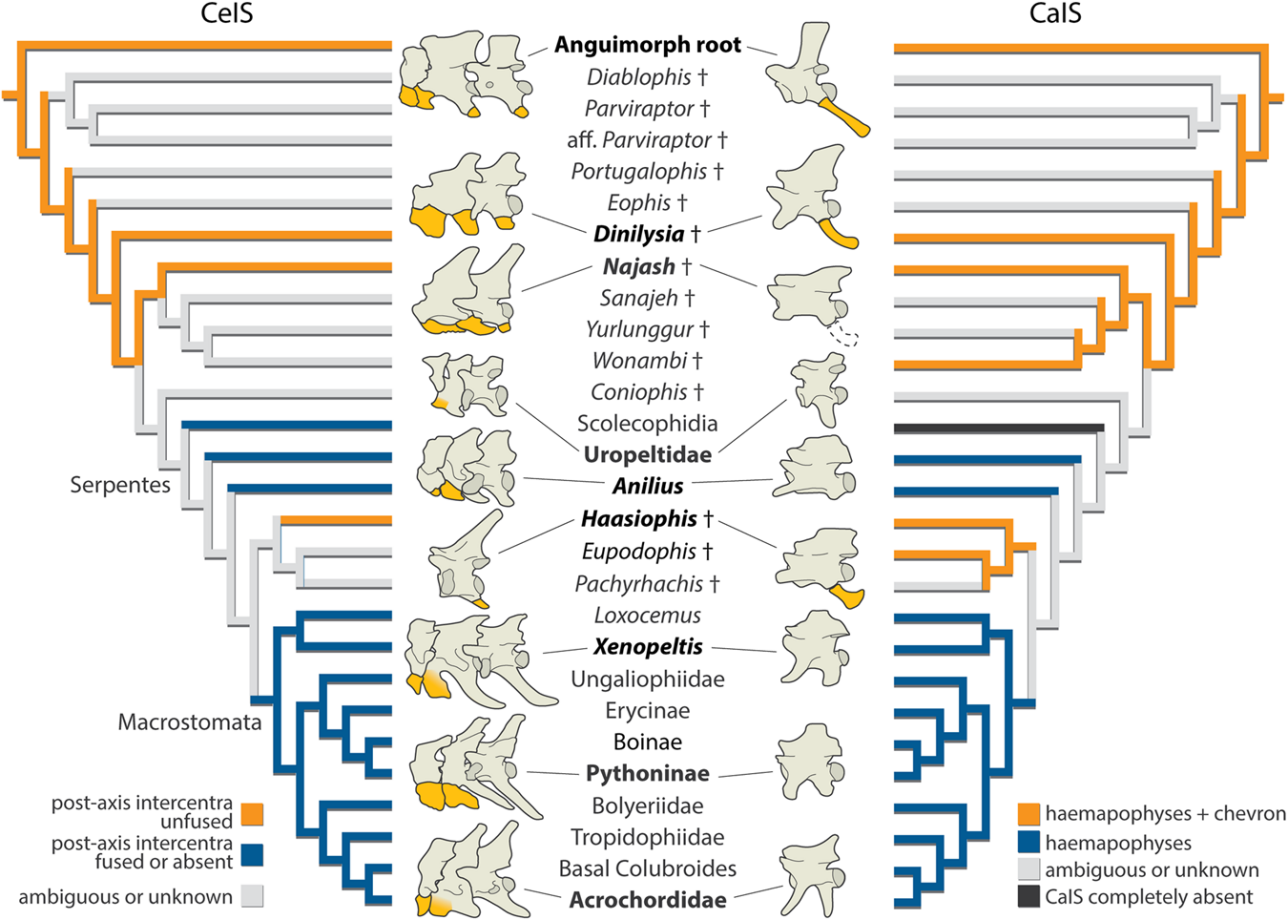
Phylogenetic analysis, single MPT of 510 steps with mapping of the presence and absence of free intercentra through snake evolution (the taxa illustrated are the same as in Fig. 1, supports and ancestral character reconstructions on Supplementary Information).

In order to assess the evolution of the CeIS and the CaIS in snakes, we performed phylogenetic analyses derived from a recently published dataset^5^, with the addition of an extra character and a few changes regarding the scoring of *Dinilysia patagonica* and *Najash rionegrina* (only considering the holotype) based on the new information presented here (Supplementary Information). We analyzed this data set using both maximum parsimony and Bayesian inference, with the results from maximum parsimony (Fig. 4) being almost identical to those of the previous study^5^. Both analyses indicate agreement on placing *Dinilysia* and *Najash* outside crown-group snakes and in having simoliophiids further crownward as early diverging macrostomatans (Figs 4 and S1). This finding is consistent with most recent phylogenetic hypotheses (e.g.^18,43^).

The morphology of the CeIS and the CaIS in *Dinilysia*, *Najash* and madtsoiids indicates that the combination exhibited by some non-ophidian squamates of unfused cervical and caudal intercentra, articulating with distinct hypapophyses or haemapophyses respectively, defines the plesiomorphic condition for ophidians. The mapping of these structures onto our re-analyses of early snake relationships further supports the hypothesis that in the CaIS, the haemapophyses of extant snakes are homologous to those of other fossil snakes, and to those of fossil and extant lizards. However, the chevron bones appear to be lost in modern snakes, but not in the Mesozoic fossil snakes highlighted here and in Palci *et al*.^11^. In the CeIS the hypapophysis of modern snakes is the homolog of the non-ophidian squamate hypapophyses, and unless a free intercentrum is present that is then observed to fuse to the hypapophysis (by undisputed morphological or developmental data), it cannot be concluded that an intercentrum is actually present beyond the atlas and axis vertebral intercentra. These results agree with the proposed loss of post atlas-axis intercentra (cervical and caudal) in modern snakes^29^, but not all snakes as we made clear from an important number of fossil snakes.

All the fossil snakes that present unfused intercentra (either directly observed or inferred) in both the caudal region and post-atlas-axis anterior trunk region (*Dinilysia*, *Najash*, madtsoiid snakes, and *Haasiophis*^8,9,11^), lie outside crown-Macrostomata and so the presence of intercentra does not contradict embryological evidence^29^. Strict adherence to the retrieved (Figs 4, S1) and commonly held (e.g.^18,43^, but see^11^ for the alternative possibility of a more basal position for simoliophiids) phylogenetic scheme would hold that the fossil evidence indicates that post-axial intercentra were lost prior to the evolution of crown-group Serpentes, but intercentra were reacquired (a reversal) in simoliophiids (Figs S5, S6). A strict reading would again be supported by the fact that scolecophidians have anterior hypapophyses, but a complete absence of caudal intercentra can be inferred since they have significantly shortened tails (<20 vertebrae) and they lack haemapophyses altogether^7,26^; basal alethinophidians such as *Anilius* have cervical hypapophyses and caudal haemapophyses without free intercentra, and uropeltids have both cervical and caudal hypapophyses.

However, we recommend a more cautious approach to such strict readings of phylogenies, particularly when ancient fossil taxa are nesting well within the crown (e.g., simoliophiids in this case). In the case of our phylogeny (Fig. 4), uropeltids, scolecophidians and anilioids are basal to simoliophiids, which are known from ~95 million year old fossil remains, and the clade they are a part of has no modern representatives. In this and other phylogenies, uropeltids, scolecophidians and anilioids are members of clades that are basal to simoliophiids (e.g.^5,18,43^); but it must be remembered, these living snake species are not early evolving snake species, but rather extant (and thus recently diverging) members of their respective early evolving clades. They are 95 million years younger than simolophiids and if their phylogenetic position is accurate, then their 95 million year old clade members, currently unrepresented by fossil remains, could show one of two conditions, either the possession of intercentra in both the cervical and caudal region, as do simoliophiids, or, they might show a condition supporting the strict reading of the phylogeny as noted above.

We would thus suggest that a more relaxed reading of Fig. 4 is possible. Even if it is not for the moment, empirical, the “relaxed reading” of Fig. 4 becomes predictive and could thus be supported by either new fossil material (e.g. fossils of early anilioids or uropeltids), or a revised phylogenetic hypothesis (such as recovering simoliophiids at a more basal position). Instead of single loss of a feature, with reacquisition or re-evolution^42^ as the primary explanations for such phylogenetic patterns of morphological change, we consider as a valuable prediction, if not alternative, that there were multiple independent events of loss of intercentra in all these non-macrostomatan groups and in derived macrostomatans. In this model, simoliophiids are the best-known fossil group at the base of macrostomatans and demonstrate the plesiomorphic condition for that clade circa 95 million years ago. This is consistent with a similar scenario proposed for the presence of well-developed hindlimbs in these snakes.

## Methods

### Described specimens

#### Dinilysia patagonica

MACN-RN-1016 (Museo Argentino de Ciencias Naturales “Bernandino Rivadavia”, Río Negro Collections, Buenos Aires, Argentina), collected in 1994–95 by a team headed by José F. Bonaparte, in the Tripailao Farm Locality, located near Paso Córdova, Río Negro province, Argentina^8^; MACN-PV-N 116 (Museo Argentino de Ciencias Naturales “Bernandino Rivadavia”, Paleontología de Vertebrados-Neuquén Collections, Buenos Aires, Argentina), that comes from the vicinity of the Universidad Nacional del Comahue, in Neuquén City, Neuquén Province, Argentina^44^; MPCA-PV 527 (Museo Provincial Carlos Ameghino, Paleontología de Vertebrados Collections, Río Negro). *Najash rionegrina*: MPCA-PV 391.

### Phylogenetic analyses

A maximum parsimony analysis was conducted in TNT v. 1.5-beta^45^ using 100 tree replicates obtained by random addition sequence (RAS), and searching for new tree topologies with tree bisection and reconnection (TBR), saving 100 trees per replication. A single most parsimonious tree (MPT) was obtained with 510 steps.

Bayesian inference analysis was conducted using Mr. Bayes v. 3.2.6^46,47^ using the MkV model for morphological data^47^ and with rate variation across characters sampled from a gamma distribution. Each analysis was performed with two independent runs of 1 × 10^7^ generations each, with eight chains per run and four swaps attempted per swapping generation. The relative burn-in fraction was set to 50% and the chains were sampled every 1000 generations. The temperature parameter for the four chains in each independent run was set to 0.04. Convergence of independent runs was assessed through the average standard deviation of split frequencies (ASDSF < 0.01) and potential scale reduction factors (PSRF ≈ 1 for all parameters) calculated at the end of the Bayesian runs. We used Tracer v. 1.6^48^ software to determine whether the runs reached stationary phase and to ensure that the effective sample size (ESS) for each parameter was greater than 200.

## Supplementary information

Supplementary Information
