# Supplementary material for: The evolution of the axial skeleton intercentrum system in snakes revealed by new data from the Cretaceous snakes Dinilysia and Najash
Source: Sci Rep. 2019 Feb 4;9:1276. doi: 10.1038/s41598-018-36979-9 (PMC6362196; doi:10.1038/s41598-018-36979-9)
Supplement: Supplementary file 1 — Supplementary Information [file 41598_2018_36979_MOESM1_ESM.pdf]

The evolution of the axial skeleton intercentrum system in snakes revealed by new data from the Cretaceous snakes *Dinilysia* and *Najash*

Fernando F. Garberoglio, Raúl O. Gómez, Tiago R. Simões, Michael W. Caldwell and Sebastián Apesteguía

Supplementary Information

1. List of Institutional abbreviations:

FMNH, The Field Museum of Natural History, Chicago, U.S.A.; **HUJ-Pal,** Hebrew University of Jerusalem, Palaeontology Collections, Jerusalem, Israel; **MACN-RN**, Museo Argentino de Ciencias Naturales "Bernandino Rivadavia", Río Negro Collections, Buenos Aires, Argentina; **MACN-PV-N**, Museo Argentino de Ciencias Naturales "Bernandino Rivadavia", Paleontología de Vertebrados-Neuquén Collections, Buenos Aires, Argentina; **MCZ**, Museum of Comparative Zoology, Massachusetts, U.S.A.; **MPCA-PV**, Museo Provincial Carlos Ameghino, Paleontología de Vertebrados Collections, Río Negro, Argentina; **UAMZ**, University of Alberta Museum of Zoology, Alberta, Canada; **USNM**, National Museum of Natural History, Washington, D.C., U.S.A; **ZFMK**, Zoologisches Forschungsmuseum Alexander Koenig, Bonn, Germany.

2. Supplementary Figures

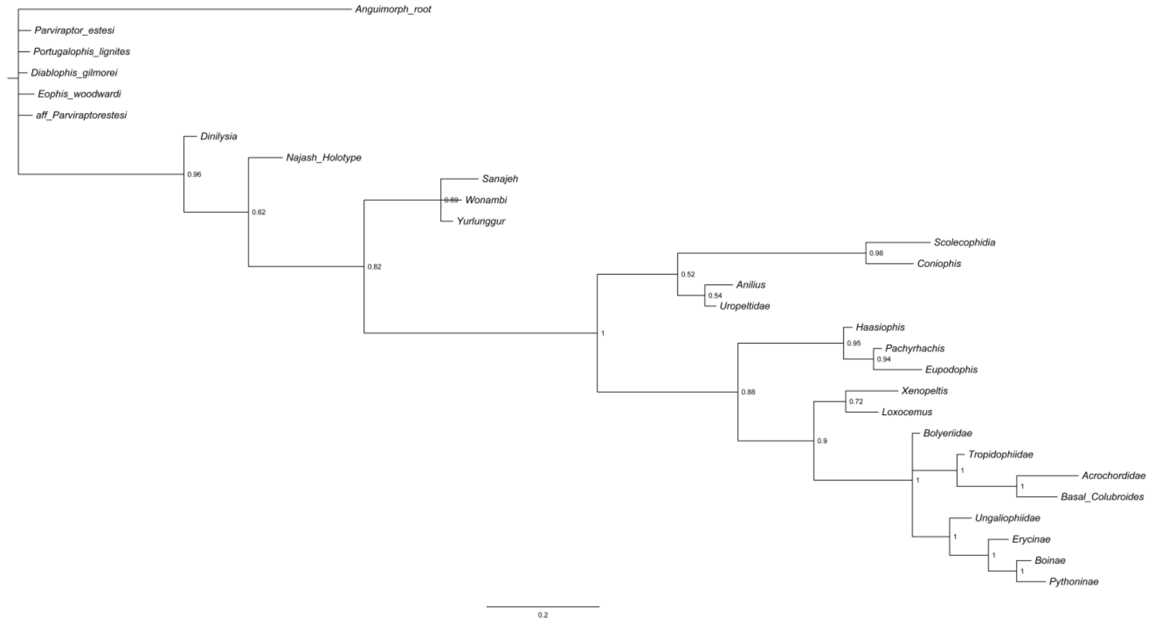

Figure S1. Majority rule consensus of Bayesian Inference analysis. Number indicates posterior probabilities.

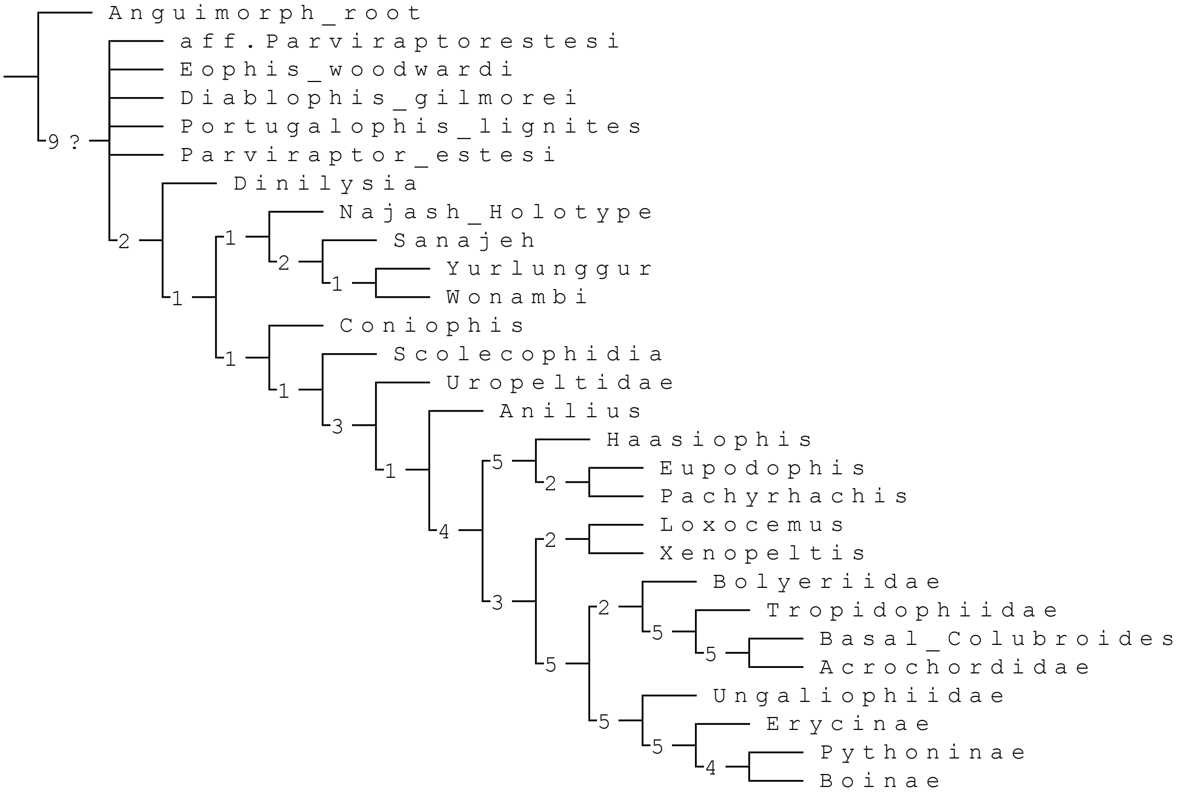

Figure S2. Bremer supports for the MPT from the maximum parsimony analyses (from

675 trees, cut = 0).

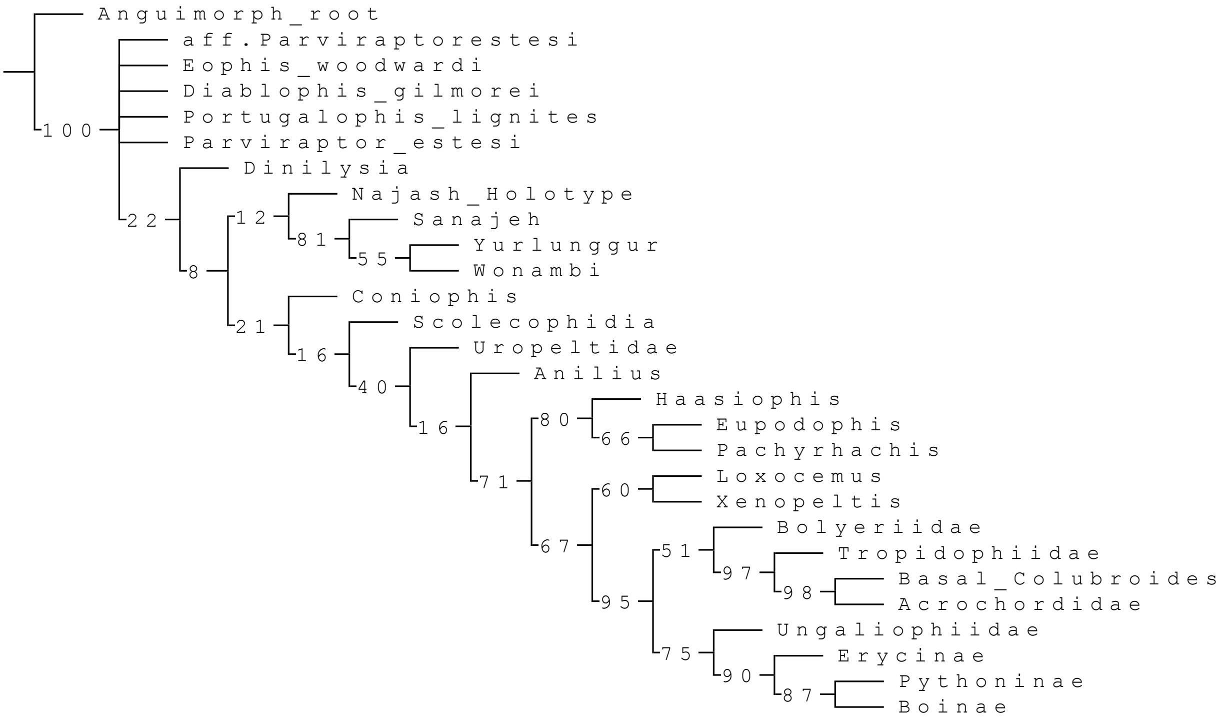

Figure S3. Standard bootstrap support for the MPT from the maximum parsimony analyses (1000 replicates, cut = 50).

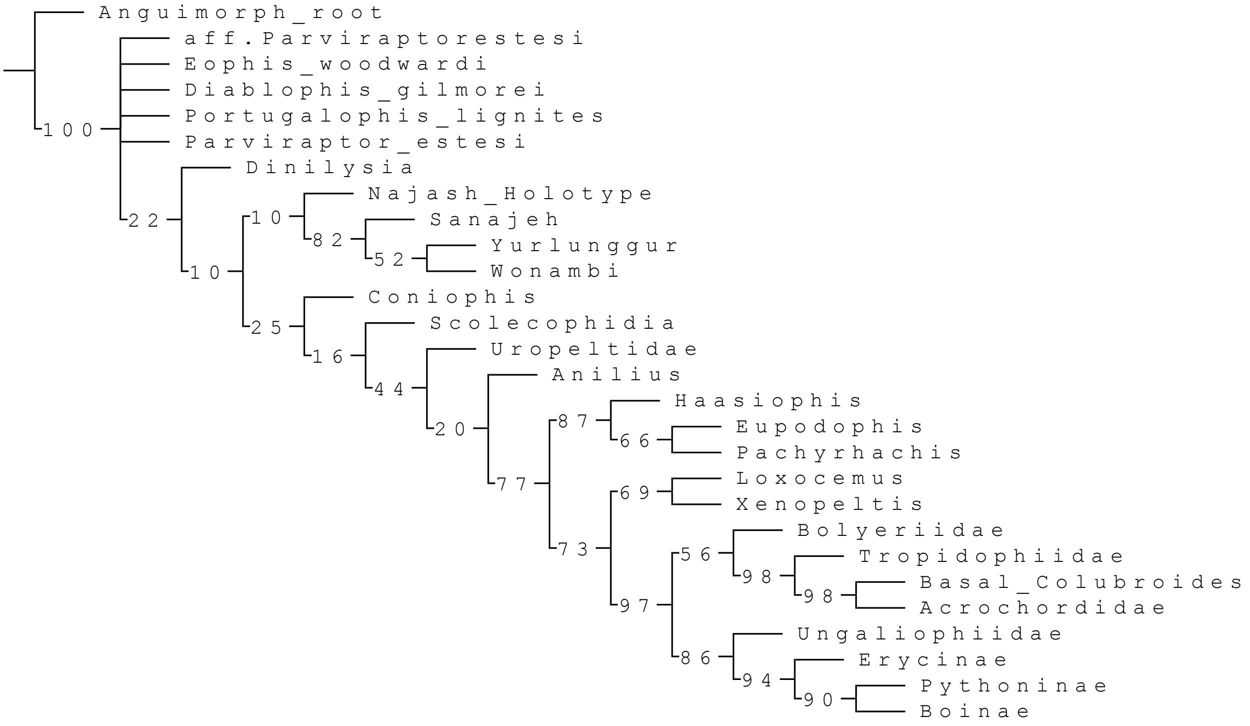

Figure S4. Jacknife support for the MPT from the maximum parsimony analyses (1000 replicates, cut = 50).

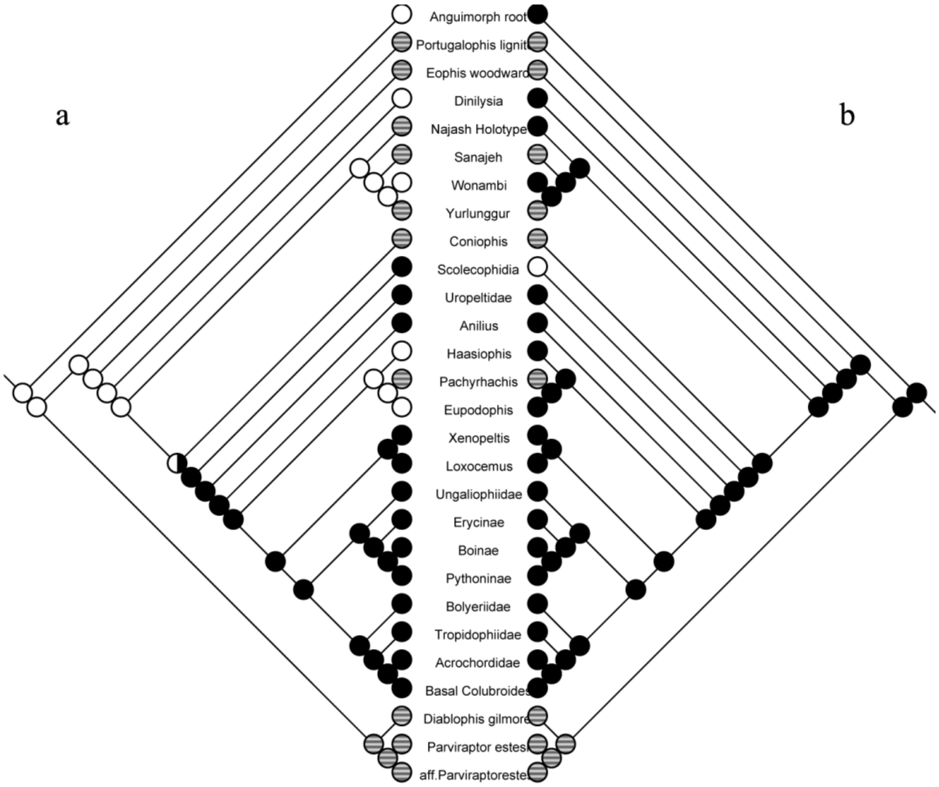

Figure S5. Ancestral character state reconstruction for the CaIS, based on the MPT. a) Presence of intercentra, white circles indicates present, black absent, and striped is unknown. b) Presence of haemapophyses, white circles indicate absent, black present, and striped is unknown.

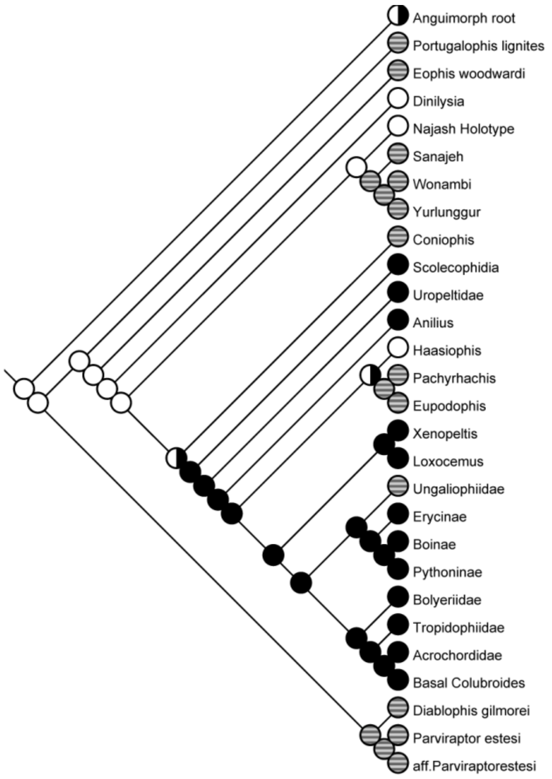

Figure S6. Ancestral character state reconstruction for the CeIS, based on the MPT. Presence of unfused intercentra in precloacal vertebrae posterior to the axis, white circles indicates present, black absent, and striped is unknown.

3. Supplementary Data

Data matrix of Caldwell et al.[1] with the addition of a new character - Character 238: Unfused intercentra in precloacal vertebrae posterior to the axis: present (0); absent (1) - and with character-state scorings corrections for *Dinilysia* and the holotype of *Najash*:

xread

238 28

Anguimorph_root

00001[0 1]0001?00[0 3]0?0000010000000[0 1]0000010000?00010000-000000000?000000001000101000000?100?0000?0000000-0000000000000000001000[0 1][0 1]0000000?00[0 1]0000000000000000[0 1]0000?0000000000000000000000000000[0 1]0000000[0 1]0-0000000000000000100000-0000000000000000[0 1]000[0 1]

Najash_Holotype

11??1???
?????????????????????0??????101011?100011100001?1?000110???????????1???????????????
?????????????????????????????????????10???0??20????1?????????01111100

Scolecophidia

11[1 2]1101110?1010??000??00???0000211[0 1]??000?11?11?[0 1]1?000[0 1]0[0 1]020---1[0
1]0?01000000111112100100000?0?02000010111[0 1]010000000110001120[0 1][0 1]10[0 1]01[1
2]11011111[0 1]11101??1110[0 1]0[0 1][0 1]1000110000011110101O?0011112111000011--1[0
1]0--1-10000111?0[0 1]0102[0 2]000121110[0 2]10[0 1]111001001

Dinilysia

112?111??1?100??????0?01021000001001001001001101000000000?01?0000000000000010001101
010110000001[0 1]100000?11100120?00001010101[0 1]01000[0
1]010?101?1???????001000000001???????0001120200100111111111110100001-1100010000-
0110101010110001110011101101111110

Sanajeh

111??????????????????????2??????1?????0??10?01020?00?0?0??1???1??????1????????01?111
1100??0???12?10????1?12?1????1??010???1?0111???001?1???????????11?????????1????????
???01?0????????????????????????????0?1??1??0?????1???1??????????1111??1?

Wonambi

112?111?????0?????????0??2?????1?????01011001112001 1?010101?011101?0?1????????0101
11100000000111211 01?111?1201?0??101010?111?01111??001?1????120????11?0???1??01???0
0?11202011??1110111111 0100?0?????10?-00110?111011111110011???1110101111111?

Yurlunggur

112111101??103??0?01000001?00?1110010011010??1020001?0101010011101 0011?010????01
011?1?000?001112110111 11?12?0????1??010111100111???001[0
1]1???????10?011001??1??0111000011202011 0?11101??1110??0001?11010?000110???10??1?
1?????1?00??????1111111?

Anilius

1110111000010020000100101110001111 1?1001010?11010000000100110011111000000 1011110
1000100110001021000011010111012001111011110101010111101111111110111011101111100
0110100112120010011111121111110111-110001110000111111000201101??10??101111001101

Uropeltidae

11111[0 1][0 1]0[0 1][0 1]010[0 1]100[0 1]0100100110001111[0 1]01001010?110[1 2]00000[0
1][0 1]00110010111000[0 1]00101111[0 1]1000100010001021[0 1][0 1]0[0 1][0 1]111011[1
2]0120011110111101010101110111111110111011101111110[0 1]0110[0 1]001121[0 2][0
1]01001[0 1][0 1]1112111[0 1]11[0 1]11--1101011101001111[0 1]00[0 1][0 1]20[0 1]001111[0
1][0 1]2101111001[0 1]01

Pachyrhachis

112??11?1??10?????????00???00???11011??0?11??112???0101??11??????10111?111?20?1???
???????1??????????10???120110001??0101111?001?121111?11011110???????0???1????110?0
01???2??1???1?1???1?1????1-?-1011????????111?0?1???1?111?????1?01111021?1?

Haasiophis

112?111?1??10???????0?00???00???110110?1?11?01121?00101??11?11??11?111?011????1???
?0????11?????1?0??10???1201100010101011111?10??121?11011011110???????01111???11?0?0
011??[1 2]101?0??1????1??????10?-1011????????111?0????20???111101?101111?2??10

Eupodophis

112?011?1??10?????????????1???????1011????1???1101???1011?11????2?101???1?1????1????
?????????????1????1????12011000101000111?00??0?111111011?10???????0???1???11??????
1??????????1?1??1??????1????011?????????1?1??????1???????1????0?11112??1?

Xenopeltis

1120011011010220010100110310001111??0010111020200001011011001111111111001000111
10101000101010111000110012120120111110111101110101211010111----
11111111011111011111011112111101110?110?2111-11110-
0111111100000111101010201111211112111111001101

Loxocemus

112001101101021001010011021000111000100101110202001010110110011111111110010121011
1010100[0
1]1010101110111100121201201111101111011101012110101111?201111111011111101111100
1112111011111111?21111110102010111110100011111111120110111111110111101111

Erycinae

1121011111011[0 1]1011011011012[0 1]10111[0 1]0[0 1]1100111[0 1][1 2]211101010[0
1]1011111121111[0 1]1001112001201110[0 1]1011[0 1]?11[1 2]0[0 1]011[0
1]0101211201111101111010011012110101111122011111110111111021110001[1 2]11101[0
1]11?111?211111101[0 1]-1101101211001111110112111[0 1]121101[1 2]101110011111

Ungaliophiidae

11210110110110201101101[0 1]0120101111001100111?021[0 2]1000101101111112[0
1]1110100111200120101000101110110001110O1?1211?1-
21110111101001101211010111112201111111101??11[0
1]011110001121111111111111121111111011-
0101101211001111111111211111211112101111001101?

Boinae

1121011[0 1]11011[0 1]2011011011[0 1]1211011110111[0 1]0111122121011101[1
2]0111111201111100111200121111[0 1]00[0 1]1111011120101100121211201111101111[0
1]10011012110101111122011111101111110211101011212111111111112111111110-
11011012110011111101121111111101210111101111111

Pythoninae

112[0 1]011[0 1]11010020010110111121101110011100111?02121011101201111[0
1]10211111100111200121111100[0 1]101[0 1]00112[0
1]101100121211201111011110100110121101011111220111111011111102111010112121111
1111111211111010211011012110011111101121111111111110111111111

Tropidophiidae

11210111110113200101001101101111110111001111?021011101010011101121111010011120O1
201110111011101110011100 1?12112012111111111100110111010111112 2[0
1]1111111111111110111100011211111111?111?2111111110-
010110111000111110111122111121101211111100 1101

Bolyeriidae

11210110110113200101001101101011110111001111?0212001010110111011211110100111201 1
201110001011??1100011100121211201111011111100110121101011 1----
1111111011111101111000112111111111?111?2111110110-0101101110001111111111120[0
1]1112110121[0 1]1111001101

Acrochordidae

11210111101113100112111112001111101111 0?11?0212111010100111011201111102112001
???01011101110111001110010120 1?1-21111111111[0 1]01101211010[0 1]11----
1111111111??11101111000111101110 01?111?2111110110-
11011011000001101 0?1111210?121101211111101 1101

Basal_Colubroides

112101101[0 1][0 1]113110112111[0 1]0[0 2][0 1][0 1]111111[0 1]01100111?021[0 1 2][0
1]110101001110102O111[0 1]11021130012111101110 11[0 1][0 1]1[0 1]0001110[0 1]101211-1-

211111110110[0 1]1101211010111----1111111111??1110111100011211111111?111?21110-1110-11011011000011111011102211012110121110110011111

Coniophis

111?1???????????????????????????????????????0??????????????????????????????????????
?????????????????????????????0001201?10[0
1]01??10????????????????????1??0???????????????????0??????????????????????????11???
?1?10?????????????0100100?

Parviraptor_estesi

112?1?1????????????????????????????????????10101??????????????????????????????????
??1??0?????????????
????11??012???0-???

Portugalophis_lignites

112?1?1????????????????????????????????????10101??????????????????????????????????
??????????????????????0???1??00????????????
????11?0012???0-1?????????????????101??0??0??????????????????????

Diablophis_gilmorei

112?1??????????????????????????????????????1??01??????????????????????????????????
?????????????????????0????????1000?0?001????10????????????????????1??0?????????????
????1???012???0-??????????????????101??0??0???????????????000110?

Eophis_woodwardi

1???1??
??????????????????1??0???1?????????????????
?????????????????????????????????101?00??0??????????????????????

aff.Parviraptorestesi

?????????????????????0???????0??0??
??????????????????????????????1000?0?001????10???????????????1?????????????????????
??000?10?

;

4. Supplementary references

1. Caldwell, M.W., Nydam, R.L., Palci, A. & Apesteguía, S. The oldest known snakes from the Middle Jurassic-Lower Cretaceous provide insight on snake evolution. *Nat. commun.* **6 (5996)**, 1–11 (2015).
